# Evidence of Tolerance to Silica-Based Desiccant Dusts in a Pyrethroid-Resistant Strain of *Cimex lectularius* (Hemiptera: Cimicidae)

**DOI:** 10.3390/insects7040074

**Published:** 2016-12-09

**Authors:** David G. Lilly, Cameron E. Webb, Stephen L. Doggett

**Affiliations:** 1 Department of Medical Entomology, University of Sydney, Westmead Hospital, Westmead, NSW 2145, Australia; dlil7680@uni.sydney.edu.au (D.G.L.); cameron.webb@health.nsw.gov.au (C.E.W.); 2 Department of Medical Entomology, Pathology West—ICPMR Westmead, Westmead Hospital, Westmead, NSW 2145, Australia

**Keywords:** bed bugs, *Cimex*, insecticide resistance

## Abstract

Insecticide resistance in bed bugs (*Cimex lectularius* and *Cimex hemipterus*) has become widespread, which has necessitated the development of new IPM (Integrated Pest Management) strategies and products for the eradication of infestations. Two promising options are the diatomaceous earth and silica gel-based desiccant dusts, both of which induce dehydration and eventual death upon bed bugs exposed to these products. However, the impact of underlying mechanisms that confer resistance to insecticides, such as cuticle thickening, on the performance of these dusts has yet to be determined. In the present study, two desiccant dusts, CimeXa Insecticide Dust (silica gel) and Bed Bug Killer Powder (diatomaceous earth) were evaluated against two strains of *C. lectularius*; one highly pyrethroid-resistant and one insecticide-susceptible. Label-rate doses of both products produced 100% mortality in both strains, albeit over dissimilar time-frames (3–4 days with CimeXa vs. 14 days with Bed Bug Killer). Sub-label rate exposure to CimeXa indicated that the pyrethroid-resistant strain possessed a degree of tolerance to this product, surviving 50% longer than the susceptible strain. This is the first study to suggest that mechanisms conferring resistance to pyrethroids, such as cuticular thickening, may have potential secondary impacts on non-synthetic insecticides, including desiccant dusts, which target the bed bug’s cuticle.

## 1. Introduction

The re-emergence of bed bugs, namely both the common species, *Cimex lectularius* L., and the tropical species, *Cimex hemipterus* (F.) (Hemiptera: Cimicidae), as a public health pest over the last 15 years has presented a unique challenge from both a clinical and management perspective [1,2], and has necessitated a multi-disciplinary approach in an attempt to develop best practice control options [3,4]. Key amongst these has been a response to the growing global evidence that most field bed bug populations have developed resistance to multiple insecticide groups [5,6,7,8,9,10] and, in particular, that they have become highly resistant to pyrethroid insecticides [11,12,13,14,15,16,17,18,19,20,21,22,23]. Despite this, and due to a general scarcity of alternative control options [24], when faced with a bed bug infestation both pest management professionals (PMPs) and residents of infested properties continue to apply insecticides in attempts to achieve control [24,25,26,27,28]. Several different formulations of insecticides are available, although liquid sprays (96%) and dusts (93%) were found to be to most commonly selected insecticidal formulations used by US-based PMPs when surveyed in 2015 [28].

Insecticidal dusts would normally be recommended for only limited application to areas such as voids, cavities, behind electrical plates, under carpet or skirting boards, and other non-obvious locations. This is typically in an effort to both control the infestation but also to eliminate harborage options and the potential spread of the infestation [3], as it is well known that bed bugs can migrate from one unit or apartment to the adjacent dwellings in multi-occupancy buildings [24,25]. However, in recent times, dusts have also been proposed for, and used, around the interior of properties (such as around the perimeter of bedrooms and lounge rooms, on bed frames and sofas, or as a fabric-treated “dust band” on furniture legs) as a low-cost, long-term residual treatment option applicable particularly in properties housing the financially or socially disadvantaged [27,29,30,31]. Such a modified application of dusts for bed bug control has the potential to achieve high levels of population reduction (typically in the vicinity of 95%–98%), but rarely complete elimination [29,30,31].

Despite being in use for many decades against cockroaches, drywood termites, mites, and stored product beetles and moths [12,32,33,34,35,36], silicon dioxide-based (SiO_2_) desiccant dusts (both naturally-occurring diatomaceous earth and synthetically-manufactured silica gel) are a relatively new option available to PMPs for bed bug control. These dusts are regarded as a popular treatment option due to characteristics of low mammalian toxicity, long residual life (in dry environments), and an orthodox view that as a result of the non-chemical mode of action they are non-selective for physiological-derived resistance [24,33,37]. Both diatomaceous earth and silica gel-based dusts will absorb lipids from the insect’s cuticle, leading to eventual dehydration and death [32,34,38,39]. Diatomaceous earth is both abrasive and sorptive [32,40], however, against various stored product pests it has been found to be significantly less effective than non-abrasive silica gels, even when used in higher doses [32].

Both forms of desiccant dust possess discernable efficacy against bed bugs [24,41,42,43,44,45,46,47] although results have been variable depending on the product tested and bed bug strain(s) used. Diatomaceous earth has been found to be effective, under laboratory conditions, at application rates between 1–8 g/m^2^, achieving 100% mortality against adults over the course of 9–15 days and 99% mortality against nymphs after 2 days [24]. Contrastingly, two studies (one field trial-based study and a second, double-blind laboratory study) have otherwise presented evidence that diatomaceous earth may be largely ineffective over longer monitoring periods [44,48], although this may be due to environmental factors such as humidity or the physical properties of the diatoms and how the product has been manufactured [34,38]. When compared, silica gel appears faster-acting with 100% mortality achieved typically within 24–72 h compared to >90% mortality after 10 days for diatomaceous earth [43,45,46]. The addition of bed bug alarm pheromones improves efficacy [42], as does employing CO_2_ as an attractant to increase contact with dust residues [49] although, conversely, the addition of insecticidal compounds such as 1% pyrethrins otherwise appears to have negligible impact [12].

To date, the effect of any insecticide resistance conferring mechanisms on dust performance has only been superficially investigated. Most notably, two studies have reported that pyrethroid-resistant strains of *C. lectularius* took longer than susceptible strains to succumb to desiccant-based dusts [12,46]. Given both studies employed dusts with an insecticide component, the authors postulate that differences in each strain’s response to the insecticide component most likely explains the observed variation. However, Anderson and Cowles [12] also note that “pyrethroid-resistant bed bugs have adaptive changes to their integument that could provide some protection against desiccation”.

Cuticle thickening has most recently been confirmed as a mechanism conferring insecticide resistance in an Australian strain of *C. lectularius*, with the most resistant bugs (themselves selected from a highly pyrethroid-resistant strain) possessing a cuticle that is up to 15.3% thicker than that of a susceptible *C. lectularius* strain and 16% thicker than less-resistant bugs from within the same strain when measured under scanning electron microscope [50]. In addition, research on *C. lectularius* strains collected in the US has determined that pyrethroid-resistant bed bugs over-express genes responsible for cuticle protein development [16], and that other mechanisms of insecticide resistance (such as metabolic detoxification enzymes and *kdr*-type target site mutations) are concentrated in the epidermal layer of the bed bug’s integument [19]. The impact of the concentration of such resistance mechanisms all within the insect’s integument against insecticides has yet to be fully quantified, and any impact on the efficacy of products containing desiccant dusts is wholly unknown. Thus, in lieu of these research findings, it is the aim of this paper to assess the efficacy of two desiccant dusts (one diatomaceous earth-based, one silica gel-based) against a highly pyrethroid-resistant strain of *C. lectularius* that possesses a known profile of *kdr*-type target-site insensitivity, esterase-derived metabolic detoxification, and thickened cuticle, compared with a susceptible *C. lectularius* strain.

## 2. Materials and Methods

Storage and culturing of the bed bug strains were conducted as approved by the Westmead Hospital Animal Ethics Committee (WHAEC Protocol N° 2002) and in accordance with NSW Animal Research Review Panel (ARRP) Guidelines for the Housing of Rats in Scientific Institutions.

### 2.1. Experimental Insects

Resistant strain *Cimex lectularius* specimens were collected from a single, field infestation from a domestic house in the suburb of Parramatta, New South Wales, Australia, in December 2012. The specimens were examined for species [51] and, thereafter, used to establish a colony in the departmental insectary maintained at 25 °C (±1 °C) and 75% (±10%) RH with a photoperiod of 12:12 h (L:D) [5]. No insecticide selection was undertaken.

This strain has been extensively profiled for potential mechanisms conferring pyrethroid resistance, and has been found to possess haplotype B (homogeneous L925I) *kdr*-type resistance [13], hydrolytic esterase-derived metabolic detoxification [52], and cuticle thickening [50]. Exposure to a d-allethrin based rapid resistance field assay elicits no statistically significant levels of knockdown when compared to an untreated control [53].

An additional laboratory strain of *C. lectularius* maintained in colony since the 1960s with known susceptibility to pyrethroids (designated the “Monheim” strain [5]) was also used. The Monheim strain lacks any *kdr*-type mutation (homogeneous haplotype A) [13].

To ensure all bugs used in the experiments were of equal age, cohorts of fifth instar bugs were isolated from the stock colonies, fed to repletion, and monitored daily for appearance as adults. Once matured, the bed bugs were immediately separated by sex to limit the potential for breeding, and aged for a further 9 days without any further blood meal. Only 9-day-old, male, adult bed bugs from either strain were used in the dust exposure assays.

### 2.2. Products

Commercial samples of CimeXa Insecticide Dust (“CimeXa”, 92.1% SiO_2_ as amorphous silica, Rockwell Labs Ltd., North Kansas City, MO, USA) and Bed Bug Killer Powder (“Bed Bug Killer”, 96% diatomaceous earth, Bed Bug Barrier Pty Ltd., Saint Kilda, VIC, Australia) were purchased through Do It Yourself Pest Control Supplies (www.doyourownpestcontrol.com) and Globe Pest Solutions (www.globepestsolutions.com.au) respectively.

### 2.3. Desiccant Dust Assays

Filter paper discs (Advantac No. 2, 90 mm diameter, Advantec MFS Inc., Dublin, CA, USA) were placed in 9cm diameter Petri dishes and weighed on an analytical balance (sensitivity 0.1 mg) to determine pre-application weights. The amount of dust required was then calculated using the published label application rates of 2 oz/100 ft^2^ for CimeXa and 4 g/m^2^ for Bed Bug Killer, with each value proportioned to the area of the Petri dish (63.62 cm^2^). An additional treatment of CimeXa applied at 50% of the recommended label rate (1 oz/100 ft^2^) was included for the purposes of this study only in an attempt to prolong the advancement of mortality and was informed by pilot assay results that indicated the onset of mortality was rapid. Due to previously published research with diatomaceous earth indicating a slow mode of action even at high doses, no further doses either above or below recommended label rate were included for this product. Each dust was then applied by hand directly from the product containers according to the calculated rate and weighed to ensure the dose applied did not exceed ±5% (Table 1). Any excess dust was removed with a flat-headed probe. No spreading or brushing of the dust was undertaken, with dusts allowed to spread and/or clump according to how it was dispensed from the product package. Controls consisted of untreated filter papers in 9 cm diameter Petri dishes.

Four replicates of 10 male adult bed bugs that had been aged to 9 days old were added to each petri dish and were continuously maintained on the treated surfaces for the duration of the study. Mortality was recorded hourly for 24 h, again at 36 h, then daily for 14 days or until all bed bugs had died. Mortality was defined as the bugs not responding to gentle pressure through probing.

LT_50_ values were estimated using a Logit generalized linear model in IBM SPSS Statistic v23 for Mac (IBM Corp., Chicago, IL, USA). Cumulative mortality values at each time point were then also used to create Kaplan-Meier survival curves, with differences between the Parramatta and Monheim strain bugs within treatment groups compared by Log-rank tests.

## 3. Results

**Figure 1:**
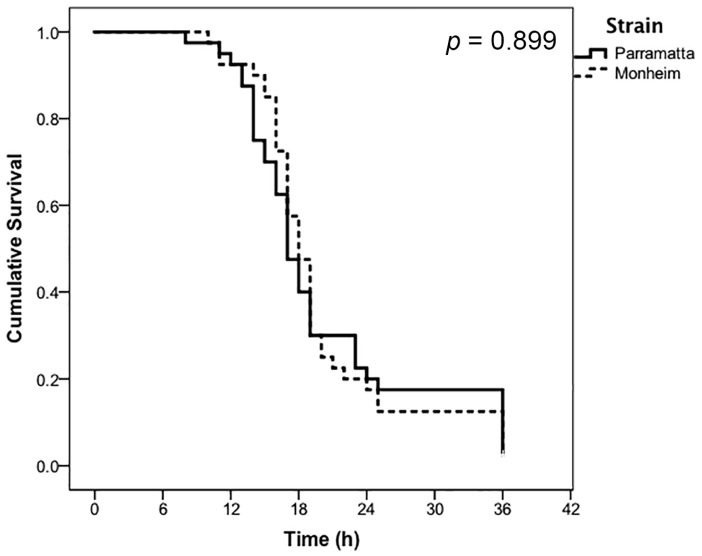
Proportional cumulative survival in response to continuous exposure to the prescribed label rate of CimeXa Insecticide Dust of Parramatta (resistant) and Monheim (susceptible) strains of *Cimex lectularius* (*n* = 40 bugs per strain, control mortality = 0%).

Adult, male bed bugs exposed to both applications rates of CimeXa dust began to succumb after more than 6 h of continuous exposure, with survival decreasing along a broadly inverse-sigmoidal manner over the resulting 36–72 h. At the prescribed label rate of CimeXa, complete mortality was achieved after 36 h for both strains of bed bugs, with no statistically significant difference (*p* = 0.899) existing between the Parramatta (resistant) and Monheim (susceptible) bugs in the rate of diminishing survival over the course of the experiment (Figure 1). No control mortality was recorded within 36 h on the untreated filter papers for either strain of *C. lectularius*.

**Figure 2:**
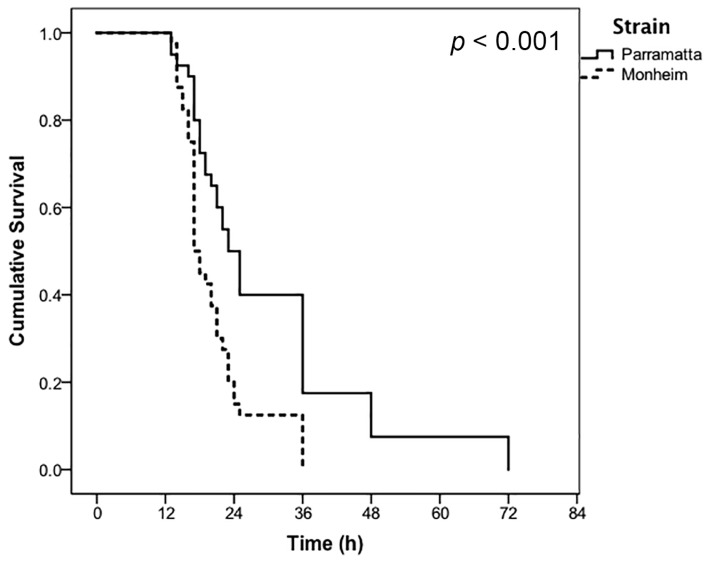
Proportional cumulative survival in response to continuous exposure to the half (0.5×) prescribed label rate of CimeXa Insecticide Dust of Parramatta (resistant) and Monheim (susceptible) strains of *Cimex lectularius* (*n* = 40 bugs per strain, Monheim (susceptible) control mortality = 0%, Parramatta (resistant) control mortality at 72 h = 2.5%)

At half of the prescribed label rate of CimeXa dust, onset of mortality began to occur after approximately 12 h of continuous exposure, however, the Parramatta (resistant) bugs ultimately took longer to succumb (Figure 2) with a statistically significantly lower rate of decrease (*p* < 0.001) compared to the Monheim (susceptible) bugs. Interestingly, Monheim (susceptible) strain bugs succumbed after 36 h regardless of the dose being applied, whereas Parramatta (resistant) strain bugs took ≈50% longer to die at half label rate (72 h) than at the prescribed label rate (36 h). No control mortality was recorded within 36 h on the untreated filter papers for the Monheim (susceptible) bed bugs, and only 2.5% was recorded with the Parramatta (resistant) bed bugs at the final 72-h monitoring point.

**Figure 3:**
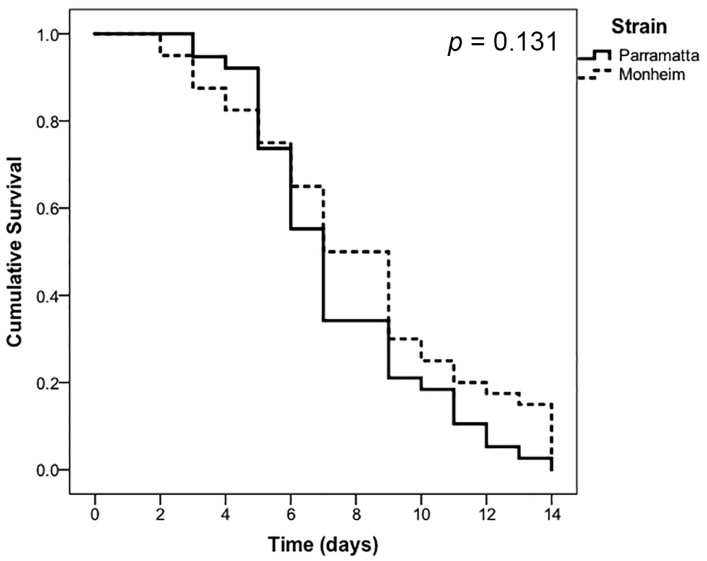
Proportional cumulative survival in response to continuous exposure to the prescribed label rate of Bed Bug Killer Powder of Parramatta (resistant) and Monheim (susceptible) strains of *Cimex lectularius* (*n* = 40 bugs per strain, control mortality at 14 days (both strains) ≤ 5%).

Bed Bug Killer proved to be a much slower acting product compared to CimeXa, with a full two weeks (14 days) required before 100% mortality was achieved in either strain (Figure 3). No statistically significant difference was observed in mortality or the rate of kill (*p* = 0.131). Control mortality in both strains did not exceed 2.5% for the first 11 days, and thereafter was ≤5% for the remainder of the experiment. Thus no Abbott’s correction was made to the treatment values [54].

Estimates of the LT_50_ values for CimeXa (Table 2) also indicated no statistically significant difference between the Parramatta (resistant) and Monheim (susceptible) bugs when exposed to the prescribed label rate, with 50% mortality expected after ≈20 h of continuous exposure. However, at half label rate of CimeXa the estimated LT_50_ value for Parramatta (resistant) bugs (30 h), was ≈50% more than that of the Monheim (susceptible) bugs (20.47 h). Failure of the 95% confidence intervals to overlap for these values indicates that this is, putatively, a statistically significant difference.

Estimates of the LT_50_ values for Bed Bug Killer (Table 3) also indicated no statistically significant difference between the Parramatta (resistant) and Monheim (susceptible) bugs when exposed to the prescribed label rate, with 50% mortality expected after ≈7.5–8 days of continuous exposure.

## 4. Discussion

Insecticide tolerance is defined as the “the natural ability of a population to withstand the toxic effect of a particular insecticide” [55] and in contrast to resistance is not, per se, a genetic change as a result of selection pressure. However, it may nonetheless be a by-product of an insect’s inherent physiological ability to detoxify an insecticide or resist penetration through the thickening of the integument. As far as current knowledge exists, desiccant dusts act exclusively on the cuticle, and the results of this study suggest that bed bugs that possess cuticle thickening as a mechanism to resist insecticides also then exhibit tolerance to desiccant dusts if exposed to sub-label rates.

Upon exposure to a silica gel product, CimeXa Insecticide Dust, applied at half the prescribed label rate, the Parramatta (resistant) bed bugs were able to survive an additional 10 h compared to the Monheim (susceptible) strain. The estimated LT_50_ for the resistant bugs was also found to be statistically significantly different, at approximately 50% more than the LT_50_ of the susceptible strain. Despite this, label rate application of the product achieved 100% mortality after 36 h, with near identical estimated LT_50_ values suggesting the product remains efficacious against resistant strains when used according to label directions. The diatomaceous earth product, Bed Bug Killer Powder, was found to be extremely slow acting compared to CimeXa, but was ultimately efficacious over the long term with no statistically significant differences observed after 14 days.

The results of this study conform to the findings of several laboratory and field studies into the effectiveness of various dust products against resistant and susceptible *C. lectularius* strains. In the United States, assays employing CimeXa against *C. lectularius* found that it was the only product of eight tested (including several other insecticide-only products, one insecticide + 95% diatomaceous earth product, and one 100% diatomaceous earth product) that resulted in 100% mortality regardless of whether bed bugs were temporarily exposed, forcibly and continuously exposed, or offered a treated/untreated harborage choice [47]. CimeXa was also found to transfer and induce mortality between treated and untreated bed bugs effectively at ratios of both 1:5 and 4:6 [47]. Separately, CimeXa was found to be highly efficacious against two pyrethroid-resistant strains and one susceptible strain of *C. lectularius*, with mortality in a laboratory-based assessment typically reaching 100% after only 24–48 h [45], as was predominantly achieved in this study.

A combination desiccant and insecticide, Drione Insecticide (1% pyrethrins, 10% piperonyl butoxide, 40% silica gel, Bayer Environmental Science, Research Triangle Park, NC, USA), was also found to work quickly (50% survival < 1 day) on hardboard and mattress-fabric surfaces, while an insecticide only dust, DeltaDust (0.05% deltamethrin, Bayer Environmental Science, Research Triangle Park, NC, USA), took significantly longer (50% survival = 3.5 days) to achieve an equivalent degree of mortality [12]. Interestingly, when tested in the same experiment, the excipient of Drione, Syloid 224 (Grace Davison, Columbia, MD, USA) performed better than Drione itself, a result explained by the fact that the end-product of Drione consists of 40% less silica that Syloid 224. The authors concluded that desiccant dusts “appear to be superior to sprayable pyrethroid products for killing bed bugs” [12].

In a similar comparative study of three desiccant dust products against US-sourced pyrethroid susceptible and resistant strains, Drione again proved efficacious against the resistant bed bugs, achieving 100% mortality in 72 h [46]. Drione also worked faster compared to a diatomaceous earth-based formulation, Mother Earth D (Whitmire Micro-Gen Research Laboratories Inc., St. Louis, MO, USA), which resulted in >90% mortality in the first 4 days, and an eventual 100% after 10 days [46]. A third, limestone-based product, NIC 325 (ACM-Texas, Fort Collins, CO, USA), proved to be ineffective against both susceptible and resistant strains. Interestingly, in the same study, Tempo Dust (1% cyfluthrin, Bayer Environmental Science, Research Triangle Park, NC, USA) proved to be the most efficacious product, killing all strains (including pyrethroid resistant strains) within 24 h. The authors theorized this could be as a result of other inert products in the formulation improving uptake of the insecticide.

In an Australian study employing diatomaceous earth, 100% mortality was also achieved with the highest dose after 9 days, and 15 days for all other doses against a pyrethroid-resistant strain of *C. lectularius* [24]. In the same study, nymphs were found to be significantly more susceptible, with 99% mortality achieved after only 2 days, although this result was unsurprising as it is known bed bug nymphs can lose water rapidly and thus are more susceptible to water stress [56]. The response of nymphs and adult females was not assessed in this study but may be an area worthy of further investigation.

Several studies have also been undertaken examining, in a mostly indirect manner, the efficaciousness of desiccant dusts as part of treatments for *C. lectularius* control in low economic or social housing [27,29,30]. When either Tempo Dust or Mother Earth D were included as part of an “Insecticides Only” or “IPM” program for the treatment of a low-income housing building in Indianapolis, IN, reductions of 33% and 44% respectively were seen after 10 weeks [30]. Both dusts were applied to many areas (such as cracks and seams around sofas and sleeping areas), although no indication was recorded by the authors as to the rationale for selecting Tempo or Mother Earth D for use, thus making direct inferences about the performance of each treatment regime and dust against each other difficult. Nonetheless, in a separate study employing a similar methodology, use of Mother Earth D, bed bug interceptor traps, and steam treatments, achieved a 97.6% (±1.6%) reduction in the number of bed bugs after 10 weeks suggesting some efficacy at population reduction over the long term [29], although again the relative contribution of each of the program components to the overall reduction is impossible to gauge. Additionally, despite the promising levels of bed bug population reduction, complete eradication was ultimately only achieved in 50% of the treated apartments, suggesting that a refinement in non-chemical control options or a secondary insecticide treatment method is needed to make the final step to elimination.

The main contrasting study to the above results achieved with diatomaceous earth concerns a field trial run in Kentucky, USA, where Mother Earth D was used exclusively in six domestic apartments, with neither chemical nor non-chemical complementary treatments undertaken over the course of an initially planned 12-week trial [48]. However, five of the six apartments had to be withdrawn prematurely from the study due to tenant dissatisfaction, and despite the intensive inspection and treatment with diatomaceous earth, average bed bug counts rose by 1%. The only apartment successfully treated involved a minor infestation, and a tenant that travelled frequently and thus was away for periods of the trial. A suspected reason for the failure was that bed bugs were only receiving “abbreviated” exposure to the dusts and were thus not receiving a lethal dose [48].

Similarly, Alpine Dust Insecticide (0.25% dinotefuran, 95% diatomaceous earth dust, Whitmire Micro-Gen Research Laboratories Inc., St. Louis, MO, USA) was evaluated in combination with Alpine Aerosol (0.5% dinotefuran, Whitmire Micro-Gen Research Laboratories, St. Louis, MO, USA) across nine *C. lectularius* infested apartments. Despite dramatically reducing the amount of insecticide applied in the course of a routine bed bug treatment, after 6 months bed bugs had only been eliminated from three apartments (as measured by visual inspection and monitoring devices), suggesting that exclusively employing a combination of neonicotinoid plus diatomaceous earth based treatments also is not, by itself, an effective solution [27]. This may be due to the recent emergence of neonicotinoid resistance in the United States [8] or, as the authors suggest, the consequence of a generally scattered bed bug distribution as a result of resident’s relocating infested belongings. However, a second study examining 6-month aged residues of Alpine Dust Insecticide found the product resulted in only 40% mortality when tested against susceptible *C. lectularius*, and negligible mortality against resistant *C. lectularius*, further suggesting this product should only be included as part of a completely integrated program of other control methods for long-term reduction or control against modern field strains of bed bugs [44].

The results of this study have important implications for future use of such desiccant dust products. Any enhancement of underlying insecticide resistance mechanisms (such as cuticle thickening) that are present within pyrethroid-resistant strains may eventually, if unmanaged, lead to increased tolerance or the possible development of cross resistance. Evidence from stored product pests has shown that, with continued exposure to desiccant dusts, some species have developed tolerance to the point of compromising product efficacy, and are thus at the threshold of evolving from tolerant to resistant [38]. For example, tolerant *Tribolium castaneum* (Herbst) (Coleoptera: Tenebrionidae) beetles have been found to both avoid diatomaceous earth dust residues down to levels as low as 75 ppm and, when forced into a treated area, will have decreased movements compared to non-tolerant strains [57]. Similarly, silica gels are known to be highly repellent to *Blatella germanica* L. (Blattodea: Blattellidae) [58]. Such adaptions, if translated to bed bugs, may complicate the development of devices such as some lethal harbourage traps which rely on the ability of bed bugs to enter traps and/or horizontally transfer small amounts of insecticidal dusts to other bed bugs, most likely during contact in a harbourage [59].

The result is also concerning when considering evidence that, under normal circumstances, pyrethroid-resistant bed bugs are more vulnerable to starvation than susceptible bugs [60], a response that is likely due to a combination of factors such as differences in size to volume ratios between resistant and susceptible bugs, and increased susceptibility to dehydration with decreasing size. Of note, however, is that the Parramatta (resistant) strain of *C. lectularius* used in this study has been found to be significantly smaller than the comparison Monheim (susceptible) strain bugs, on some body features up to 10 percent [50]. Conventional theory would dictate that the smaller the insect, the more susceptible to dehydration it becomes [61]. However, as water-loss through the cuticle accounts for >80% of overall water-loss [62,63], the potential for a thickened cuticle layer to potentially provide greater tolerance to dehydration and, thus indirectly, increased tolerance to desiccant dusts warrants further monitoring and investigation.

## 5. Conclusions

This study demonstrates that a pyrethroid-resistant strain of *C. lectularius* that possesses multiple resistance mechanisms, including cuticular thickening, is mildly tolerant to sub-label rates of silica gel-based dusts. Despite this, the development and inclusion of desiccant dusts, both diatomaceous earth and silica gel-based, for the control of bed bugs, presents as one of the more promising developments since the bed bug resurgence began. Nonetheless, preliminary evidence that resistance mechanisms derived from underlying pyrethroid-resistance may confer a natural level of tolerance to low doses of such dusts provides an early warning that any new or existing artificial selection in response to the use of insecticides will, inevitably, lead to a genetic change and the eventual manifestation of resistance.

## Figures and Tables

**Table 1:** Calculated and applied rates of CimeXa Insecticide Dust and Bed Bug Killer Powder.

Strain	Treatment	Label Rate	Replicate	Label Rate Dose (g)	Actual Dose (g)	Deviation (g)	Deviation (%)
Parramatta	CimeXa	0.5×	1	0.0194	0.0195	0.0001	0.52%
			2	0.0194	0.0195	0.0001	0.52%
			3	0.0194	0.0197	0.0003	1.55%
			4	0.0194	0.0197	0.0003	1.55%
	CimeXa	1.0×	1	0.0388	0.0387	−0.0001	−0.26%
			2	0.0388	0.0390	0.0002	0.52%
			3	0.0388	0.0390	0.0002	0.52%
			4	0.0388	0.0394	0.0006	1.55%
	Bed Bug Killer	1.0×	1	0.0254	0.0253	−0.0001	−0.39%
			2	0.0254	0.0253	−0.0001	−0.39%
			3	0.0254	0.0249	−0.0005	−1.97%
			4	0.0254	0.0254	0.0000	0.00%
Monheim	CimeXa	0.5×	1	0.0194	0.0194	0.0000	0.00%
			2	0.0194	0.0195	0.0001	0.52%
			3	0.0194	0.0192	−0.0002	−1.03%
			4	0.0194	0.0190	−0.0004	−2.06%
	CimeXa	1.0×	1	0.0388	0.0393	0.0005	1.29%
			2	0.0388	0.0386	−0.0002	−0.52%
			3	0.0388	0.0390	0.0002	0.52%
			4	0.0388	0.0383	−0.0005	−1.29%
	Bed Bug Killer	1.0×	1	0.0254	0.0255	0.0001	0.39%
			2	0.0254	0.0254	0.0000	0.00%
			3	0.0254	0.0255	0.0001	0.39%
			4	0.0254	0.0257	0.0003	1.18%

**Table 2:** LT_50_ values in hours (±95% confidence intervals) for Parramatta (resistant) and Monheim (susceptible) *Cimex lectularius* when assayed against CimeXa Insecticide Dust (at 1.0× and 0.5× label rate).

Treatment	Label Rate	Strain	LT_50_ (h)	95% CI (Lower)	95% CI (Upper)
CimeXa	0.5×	Parramatta	30.00 *	25.07	34.93
	0.5×	Monheim	20.47	18.40	22.55
	1.0×	Parramatta	20.17	17.65	22.70
	1.0×	Monheim	20.00	17.86	22.14

* Indicates statistically significant difference as determined by failure of the 95% confidence intervals to overlap.

**Table 3:** LT_50_ values in days (±95% confidence intervals) for Parramatta (resistant) and Monheim (susceptible) *Cimex lectularius* when assayed against Bed Bug Killer Powder (only tested at 1.0× label rate).

Treatment	Label Rate	Strain	LT_50_ (Days)	95% CI (Lower)	95% CI (Upper)
Bed Bug Killer	1.0×	Parramatta	7.42	6.54	8.30
	1.0×	Monheim	8.12	6.99	9.25
